# The risk-to-benefit ratio of transcatheter aortic valve implantation in specific patient cohorts: a single-centre experience

**DOI:** 10.1007/s00392-012-0426-4

**Published:** 2012-02-21

**Authors:** Miriam Puls, Tanja Viel, Bernhard C. Danner, Claudius Jacobshagen, Nils Teucher, Gunnar Hanekop, Friedrich Schöndube, Gerd Hasenfuß, Ralf G. Seipelt, Wolfgang Schillinger

**Affiliations:** 1 Department of Cardiology and Pneumology, Georg August University of Goettingen, Robert-Koch-Straße 40, 37075 Göttingen, Germany; 2 Department of Cardiovascular Surgery, Georg August University of Goettingen, Robert-Koch-Straße 40, 37075 Göttingen, Germany; 3 Department of Anaesthesiology, Georg August University of Goettingen, Göttingen, Germany

**Keywords:** Aortic valve, Aortic stenosis, Transcatheter aortic valve implantation, TAVI

## Abstract

**Background:**

Transcatheter aortic valve implantation (TAVI) has recently developed into an acceptable alternative to conventional surgery in high-risk patients. However, information on the identification of patients gaining most benefit from this procedure is still limited. The aim of this study was to evaluate safety and efficacy of TAVI in different patient cohorts.

**Methods:**

Between August 2008 and December 2010, 180 high-risk patients underwent TAVI at our institution (97 transapical and 83 transfemoral approaches). Periprocedural complications as well as mortality and incidence of MACCE during follow-up were recorded.

**Results:**

Mean age was 82 ± 5 years, and mean logistic EuroScore 27 ± 14%. In the total cohort, 30-day mortality was 8.9% and 12-month survival (according to Kaplan–Meier-analysis) 72%, with no significant differences between the two approaches. However, a significant difference in survival was obvious after stratification of patients according to logistic EuroScore mortality estimates. Survival proportions at 1 year were 62% in patients with logistic EuroScore >40%, 71% in patients with EuroScore 20–40% and 80% in octogenarians with EuroScore <20% (*P* = 0.009). Furthermore, the observed median event-free survival as an indicator for morbidity ranged between 315 days in the first, 442 days in the second and 710 days in the third group (*P* = 0.1).

**Conclusions:**

TAVI proved to be feasible with reproducible results. However, mortality and rehospitalization rates were considerably high in specific patient cohorts, suggesting that the risk-to-benefit ratio of TAVI should be validated individually. In the present study, octogenarians with logistic EuroScore <20% could be identified as candidates apparently gaining high benefit from the procedure.

## Introduction

Degenerative aortic stenosis (AS) has become the most frequent type of valvular heart disease in Europe and North America, and disease prevalence is still increasing due to the ageing population. Early valve replacement is strongly recommended in all symptomatic patients with severe AS [1]. However, open-heart surgery is considered to high risk in more than 30% of elderly patients who therefore remain untreated [2]. To address this problem, transcatheter aortic valve implantation (TAVI) was introduced initially for non-operable patients by Cribier in 2002, and has blossomed out to an alternative to conventional surgery in high-risk operable patients since then [3]. Nevertheless, information on long-term morbidity and mortality after TAVI remains limited complicating the identification of patients gaining most benefit from this procedure.

## Methods

### Study design

**Fig. 1:**
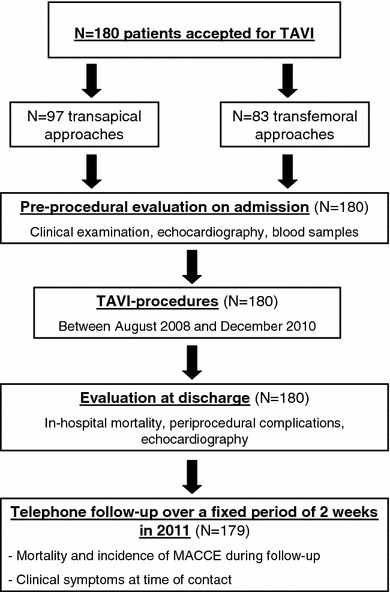
Study design

The present analysis includes the first 180 consecutive patients undergoing TAVI at our institution between August 2008 and December 2010. The aim of this retrospective study completely independent from industry was to assess safety, efficacy and benefit of the procedure. Figure 1 demonstrates the study design. Clinical examination, echocardiography and analysis of blood samples were performed on admission. At discharge, periprocedural complications and in-hospital mortality were evaluated and echocardiography was repeated. All 180 patients were followed by telephone contact over a fixed period of 2 weeks in 2011 using a standardized questionnaire to inquire clinical symptoms, further hospitalizations and cases of death. Following the patients’ or their relatives’ statements, general practitioners, cardiologists and other hospitals were contacted and medical documents were acquired to investigate the incidence of major adverse cardiovascular and cerebrovascular events (MACCE) and the causes of death during follow-up.

The study was approved by the local ethics committee, and written informed consent was obtained from all patients.

### Patient screening and eligibility

The decision to treat a patient by TAVI was made by a “heart team” consisting of an interventional cardiologist and a cardiac surgeon as suggested by common recommendations [4, 5]. Presence of severe AS was confirmed via echocardiography according to the American College of Cardiology/American Heart Association’s (ACC/AHA) valve guidelines (aortic jet velocity >4 m/s, mean gradient >40 mmHg, AVA <1.0 cm^2^). TAVI was only proposed in patients with severe AS at high risk for surgery who presented with severe symptoms (New York Heart Association, NYHA functional class ≥2). According to recommendations of the European Society of Cardiology (ESC) [4], the decision whether a patient was at high risk for surgery was made by clinical judgement in combination with the assessment of the logistic EuroScore.

### Devices and procedure

All procedures were performed by a combined team including an interventional cardiologist, a cardiac surgeon, a cardio-anaesthesiologist and an imaging specialist. Implantation procedures using the retrograde transfemoral (TF) and the antegrade transapical (TA) approach were performed as previously described [4, 6–8].

Both prostheses currently available for TAVI in Germany are used in our department. One device is the balloon-expandable Edwards Sapien valve (Edwards Lifesciences Inc., Irvine, CA, USA) available in 23 and 26 mm sizes at that time. Until May 2010, the Retroflex 3 delivery catheter (requiring 22 or 24F introducers) was used in transfemoral procedures, and arterial access as well as closure of the access site was performed surgically necessitating general anaesthesia during implantation. Afterwards, the introduction of the Novaflex delivery catheter enabled the use of smaller introducers (18/19F) and consecutively the performance of a pure percutaneous procedure in conscious sedation (closure of the access site by Prostar XL, Abbott Vascular, Chicago, IL, USA).

The second device is the self-expanding CoreValve Revalving System (Medtronic, Minneapolis, MN, USA). It was available in 26 and 29 mm sizes at that time fitting through an 18F introducer. Therefore, CoreValve implantation was performed completely percutaneously.

### Treatment strategy

Procedural approach and type of device were chosen by the “heart team” to offer the best treatment option to each individual patient. In principle, the transfemoral approach was selected in the absence of significant peripheral artery disease. Furthermore, we principally favoured the Edwards Sapien device due to the previously described lower incidence of postprocedural new pacemaker implantations. However, the CoreValve prosthesis was offered to patients whose aortic annulus was >25 mm or who were at high risk for general anaesthesia due to specific comorbidities. Since the introduction of the Novaflex catheter for transfemoral Edwards Sapien implantations allowed the performance of these procedures under conscious sedation, the number of implanted CoreValves decreased.

### Study endpoints

Recently, a consensus report from the Valve Academic Research Consortium (VARC) proposed standardized endpoint definitions to enable comparison between TAVI trials [9]. VARC definitions which are in detail described in the consensus report were adopted for the present study. Safety endpoints contained the occurrence of periprocedural myocardial infarction, TIA, stroke, bleeding complications, acute kidney injury (AKI), vascular and access-site complications, and prosthetic valve-associated complications (conduction disturbances, coronary obstruction, valve thrombosis, endocarditis). In addition, therapy-specific endpoints like unplanned use of cardiopulmonary bypass, conversion to surgical AVR, ventricular perforation, placement of a second transcatheter valve into the primary transcatheter valve (valve-in-valve), and re-intervention after the index procedure were recorded.

Concerning follow-up, all-cause mortality was defined as the primary clinical endpoint according to VARC proposals. Survival proportions were reported at 30 days, 6 months and 1 year. Furthermore, the occurrence of MACCE comprising a composite of death of any reason and hospitalization due to congestive heart failure, myocardial infarction, stroke, and aortic valve-related events (prosthesis dysfunction, re-intervention, endocarditis, thrombosis) was chosen as clinical benefit endpoint.

### Statistical analysis

For continuous variables, the Kolmogorov–Smirnov normality test was performed. Continuous variables are presented as mean ± SD or as median and interquartile range (in the absence of normality distribution) and were compared between groups using the unpaired *t* test or the Mann–Whitney test, respectively. Categorical variables are presented as absolute numbers and percentage and were compared by Pearson’s Chi-square test. A value of *P* < 0.05 was considered statistically significant. Survival analysis was performed by the Kaplan–Meier method, with patients censored as of the last date known alive. Survival proportions at 6 and 12 months were also calculated according to Kaplan–Meier-analysis. Statistical analyses were performed with graph pad prism version 4.0 and MedCalc version12.0.4.0.

## Results

### Baseline characteristics

TAVI was performed in 180 symptomatic patients (baseline characteristics demonstrated in Table 1). The total cohort was characterized by a mean age of 82 ± 5 years and a high risk for conventional surgery estimated by a mean logistic EuroScore of 27 ± 14%. In 65% of patients, the logistic EuroScore exceeded 20%. The indications for TAVI in the remaining cases were porcelain aorta, end-stage pulmonary disease, reduced life expectancy due to malignoma, and refusal of conventional surgery because of very high age (>80 years).

**Table 1:** Baseline characteristics

	Combined (*n* = 180)	Transapical (*n* = 97)	Transfemoral (*n* = 83)	*P*
Age (years)	82.1 ± 5.4	81.7 ± 5.8	82.6 ± 4.9	0.3
Sex (male)	54 (30%)	27 (28%)	27 (33%)	0.5
BMI (kg/m^2^)	26.4 ± 4.8	26.6 ± 5.3	26.2 ± 5.3	0.65
Comorbidities				
EF <35%	26 (14%)	12 (12%)	14 (17%)	0.4
Coronary artery disease	120 (67%)	64 (66%)	56 (68%)	0.8
Prior PCI	50 (28%)	26 (27%)	24 (29%)	0.8
Prior CABG	27 (15%)	18 (19%)	9 (11%)	0.09
Prior other thoracotomy	6 (3%)	2 (2%)	4 (5%)	0.3
Porcelain aorta	4 (2%)	3 (3%)	1 (1%)	0.4
Previous aortic bioprosthesis	4 (2%)	2 (2%)	2 (2%)	0.9
Peripheral vascular disease	56 (31%)	37 (38%)	19 (23%)	0.03*
Prior cerebral ischaemic event	21 (12%)	12 (12%)	9 (11%)	0.8
Chronic pulmonary disease	57 (32%)	39 (40%)	18 (28%)	0.008*
Diabetes	64 (36%)	32 (33%)	32 (39%)	0.4
GFR <60 mL/min	110 (61%)	61 (63%)	49 (59%)	0.6
GFR <30 mL/min	30 (17%)	20 (21%)	10 (12%)	0.12
Calculated surgical risk (Logistic EuroScore) (%)	26.8 ± 14.0	27.9 ± 14.9	25.6 ± 13.0	0.3
Clinical characteristics				
NYHA class III	131 (73%)	71 (73%)	60 (72%)	0.9
NYHA class IV	38 (21%)	21 (22%)	17 (21%)	0.8
Oedema	85 (47%)	46 (47%)	39 (47%)	1.0
Effusions	35 (19%)	24 (25%)	11 (13%)	0.05
Moist rales	64 (36%)	37 (38%)	27 (33%)	0.4
Syncope	46 (26%)	22 (23%)	24 (29%)	0.4

Categorical variables are presented as absolute number and percentage (in parentheses), continuous variables as mean ± SD. For comparison between TA and TF patients, the unpaired *t* test was used for continuous and the Chi-square test for categorical variables

The transapical approach was chosen in 97 cases, whereas the other 83 patients were treated transfemorally. Comparing TA and TF patients, the only statistically significant differences were the higher incidences of peripheral vascular disease (38 vs. 23%, *P* = 0.03) and chronic lung disease (40 vs. 28%, *P* = 0.008) in the TA group. Importantly, the logistic EuroScore did not differ significantly (28 ± 15% in the TA cohort vs. 26 ± 13% in the TF cohort, *P* = 0.3).

### Procedural parameters, periprocedural complications and in-hospital mortality

Procedural characteristics and incidence of specific complications are demonstrated in Tables 2 and 3. Transapical procedures were always performed under general anaesthesia, transfemoral cases in 37% under sedation and analgesia, resulting in a significantly lower median ventilation time in the TF cohort (2.8 vs. 3.6 h, *P* < 0.0001). Total procedure time, fluoroscopy time, and volumes of contrast medium were significantly higher in transfemoral procedures, whereas the time from procedure to discharge did not differ significantly between both approaches.

**Table 2:** Procedural characteristics

	Combined (*n* = 180)	Transapical (*n* = 97)	Transfemoral (*n* = 83)	*P*
Total procedure time (min)	90.1 ± 45.7	78.4 ± 38.3	104.1 ± 49.9	0.0002*
Fluoroscopy time (min)	12.1 ± 9.4	7.0 ± 4.3	16.1 ± 10.3	<0.0001*
Volume of contrast medium (mL)	116.1 ± 71.1	85.4 ± 30.7	152.3 ± 87.2	<0.0001*
Procedure to discharge (days)	13.9 ± 10.7	13.2 ± 8.9	14.8 ± 12.5	0.31
Type of valve				
Edwards Sapien (*n*)	156	97	59	–
Medtronic CoreValve (*n*)	24	NA	24	–
General anaesthesia [*n* (%)]	149 (82.8%)	97 (100%)	52 (62.7%)	<0.0001*
Conscious sedation [*n* (%)]	31 (17.2%)	0	31 (37.3%)	<0.0001*

Categorical variables are presented as absolute number and percentage (in parentheses), continuous variables as mean ± SD. For comparison between TA and TF patients, the unpaired *t* test was used for continuous and the Chi-square test for categorical variables

**Table 3:** Perioperative outcome

	Combined (*n* = 180)	Transapical (*n* = 97)	Transfemoral (*n* = 83)	*P*
Procedure-related complications				
Successful termination of procedure	174 (96.7%)	93 (95.6%)	81 (97.6%)	0.5
Conversion to surgical AVR	0	0	0	1.0
Unplanned use of cardiopulmonary bypass	5 (2.8%)	4 (4.1%)	1 (1.2%)	0.2
Ventricular perforation	1 (0.6%)	0	1 (1.2%)	0.3
“Valve-in-valve”	1 (0.6%)	1 (1.0%)	0	0.4
Coronary obstruction	2 (1.1%)	1 (1.0%)	1 (1.2%)	0.9
Re-intervention	2 (1.1%)	0	2 (2.4%)	0.1
Percutaneous	2 (1.1%)	0	2 (2.4%)	0.1
Surgical	0	0	0	–
Myocardial infarction^a^	3 (1.7%)	3 (3.1%)	0	0.1
Stroke^a^	9 (5.0%)	4 (4.1%)	5 (6.0%)	0.6
TIA^a^	2 (1.1%)	2 (2.1%)	0	0.1
Peri-interventional death (<24 h)	4 (2.2%)	2 (2.1%)	2 (2.4%)	0.9
30-day-mortality	16 (8.9%)	12 (12.4%)	4 (4.8%)	0.08
In-hospital-mortality	18 (10.0%)	12 (12.4%)	6 (7.2%)	0.25
Bleeding complications combined	94 (52.2%)	44 (45.4%)	50 (60.2%)	0.046*
Life-threatening or disabling bleeding^a^	21 (11.7%)	8 (8.2%)	13 (15.7%)	0.1
Major bleeding^a^	10 (5.6%)	3 (3.1%)	7 (8.4%)	0.1
Life-threatening + major bleeding combined	31 (17.2%)	11 (11.3%)	20 (24.1%)	0.02*
Minor bleeding^a^	63 (35.0%)	33 (34.0%)	30 (36.1%)	0.8
Cardiac tamponade	3 (1.7%)	2 (2.1%)	1 (1.2%)	0.7
Patients with RBC transfusions	87 (48.3%)	42 (43.3%)	45 (54.2%)	0.1
Number of units per transfusion	3.1 ± 2.2	2.9 ± 2.0	3.3 ± 2.4	0.5
Drop in Hb following procedure (g/dL)	2.9 ± 1.3	2.9 ± 1.2	2.8 ± 1.4	0.4
Acute kidney injury (modified RIFLE classification^a^)				
Stage 1	54 (30.0%)	24 (24.7%)	30 (36.1%)	0.1
Stage 2	41 (22.8%)	27 (27.8%)	14 (16.9%)	0.08
Stage 3	29 (16.1%)	24 (24.7%)	5 (6.0%)	0.001*
Patients requiring RRT	25 (13.9%)	21 (21.6%)	4 (4.8%)	0.001*
Patients remaining permanently dependant on RRT	2 (1.1%)	1 (1.0%)	1(1.2%)	0.9
Creatinine before procedure (mg/dL)	1.2 ± 0.6	1.3 ± 0.7	1.2 ± 0.6	0.3
Max. creatinine up to 72 h after procedure (mg/dL)	1.7 ± 1.2	2.0 ± 1.3	1.4 ± 0.7	0.0001*
Access-related complications^a^				
Major access complications	22 (12.2%)	2 (2.1%)	20 (24.1%)	<0.0001*
Unplanned surgical intervention	10 (5.6%)	1 (1.0%)	9 (10.8%)	0.004*
Unplanned percutan. intervention	6 (3.3%)	0	6 (7.2%)	0.007*
Thoracic aortic dissection	1 (0.6%)	0	1 (1.2%)	0.3
Minor access complications	6 (3.3%)	2 (2.1%)	4 (4.8%)	0.3
Prosthetic valve-associated complications				
New-onset conduction disturbances				
LBBB	12 (6.7%)	0	12 (14.5%)	0.0001*
Third degree atrioventricular block	5 (2.8%)	1 (1.0%)	4 (4.8%)	0.1
New permanent pacemaker	9 (5.0%)	1 (1.0%)	8 (9.6%)	0.008*
Supraventricular arrhythmias	29 (16.1%)	16 (16.5%)	13 (15.7%)	0.9
Valve endocarditis	5 (2.8%)	3 (3.1%)	2 (2.4%)	0.7

Categorical variables are presented as absolute number and percentage (in parentheses), continuous variables as mean ± SD. For comparison between TA and TF patients, the unpaired *t* test was used for continuous and the Chi-square test for categorical variables

^a^Definitions according to proposed endpoint definitions from the Valve Academic Research Consortium (VARC) [9]

The TAVI procedure was terminated successfully in 174 cases (96.7%). Four patients died periprocedurally (1 aortic dissection, 3 cardiogenic shocks), in one case the apex anatomy proved unsuitable for apical puncture, and during one procedure the implantation of a second device (“valve-in-valve”) became necessary (details are published in [10]). No conversion to surgical aortic valve replacement was carried out. In five patients, an unplanned use of cardiopulmonary bypass was necessary to manage hemodynamic compromise. Postprocedural myocardial infarction occurred in three persons (1.7%). Nine patients (5%) experienced major strokes with subsequent death in five cases (3 TA and 2 TF patients) contributing substantially to in-hospital mortality. Of note, the incidence of major strokes did not differ significantly between the TA and the TF cohort (4 vs. 6%, *P* = 0.6).

Access-related problems were more common in the TF group (*P* < 0.0001) with 20 TF patients (24%) experiencing major vascular complications. One of them died consequently (thoracic aortic dissection). However, we also experienced two patients (2%) with major access-complications in the TA group (1 primary failure of apex closure, 1 purulent wound infection with indication for surgical re-intervention); the first patient died on day 55.

Bleeding was a common problem following both TAVI procedures, necessitating red blood cell transfusions in 48% of TAVI-patients (mean, 3.1 ± 2.2 U per transfusion). However, relevant bleeding complications (combination of life-threatening and major bleeding) occurred more frequently after transfemoral procedures (24 vs. 11%, *P* = 0.02).

Worsening of renal function was observed more often after transapical procedures despite lower volumes of contrast medium used. Whereas baseline serum creatinine levels did not differ significantly, the maximum levels up to 72 h after the procedure were significantly higher in the transapical cohort (2.0 ± 1.3 vs. 1.4 ± 0.7 mg/dl, *P* = 0.0001), resulting in a significantly higher need for renal replacement therapy (22 vs. 5%, *P* = 0.001).

Conduction disturbances were more frequent in the TF cohort (new-onset LBBB in 14.5 vs. 0%, *P* < 0.0001), necessitating the implantation of new permanent pacemakers in 10 vs. 1% (*P* = 0.008). In transfemoral approaches, the new onset of a LBBB occurred significantly more often with the CoreValve prosthesis (46% CV vs. 2% Edwards, *P* < 0.0001), whereas the incidence of a third degree atrioventricular block was more frequent with the Edwards device (0% CV vs. 7% Edwards, *P* = 0.2). The need for new permanent pacemaker implantation was higher after CoreValve implantation (17% CV vs. 7% Edwards, *P* = 0.2), but the difference did not reach statistical significance.

In discordance with the logistic EuroScore mortality estimate of 27%, the observed 30-day-mortality was 8.9% (12.4% in the TA cohort vs. 4.8% in the TF group, *P* = 0.08) and in-hospital mortality 10.0% (12.4% in the TA cohort vs. 7.2% in the TF group, *P* = 0.25). Four patients (2.2%) died periprocedurally. The other 14 in-hospital deaths were a consequence of stroke (*n* = 5), pneumonia (*n* = 3), cardiogenic shock (*n* = 2), septic-cardiogenic shock (*n* = 1), mesenterical ischaemia (*n* = 1), aspiration (*n* = 1), and unexplained sudden death (*n* = 1).

### Echocardiographic valve performance

At discharge, a significant improvement of multiple echocardiographic parameters could be documented. We saw a reduction in transaortic mean gradient from 43.1 ± 16.9 to 10.6 ± 5.0 mmHg, an increase in mean estimated aortic valve area from 0.69 ± 0.28 to 1.6 ± 0.5 cm^2^, an improvement of mean left ventricular ejection fraction from 49.7 ± 11.8% to 53.7 ± 8.5% and a reduction of mean estimated pulmonary artery systolic pressure from 47.8 ± 14.7 to 41.5 ± 13.4 mmHg (all *P* < 0.0001). After TAVI, a mild aortic regurgitation (paravalvular in most cases) occurred frequently (48%). However, moderate AR was only observed in 7%, and severe AR was not present.

In a subgroup of 33 patients, transthoracic echocardiography was repeated 12 months postprocedure. The sustained improvement of left ventricular ejection fraction, increase in aortic valve area, and reduction in transaortic mean gradient could be confirmed, and no significant structural or hemodynamic device deterioration was observed (data not shown; extensive analyses of long-term transcatheter valve durability have previously been published [11]).

### Mid-term outcome—mortality

**Fig. 2:**
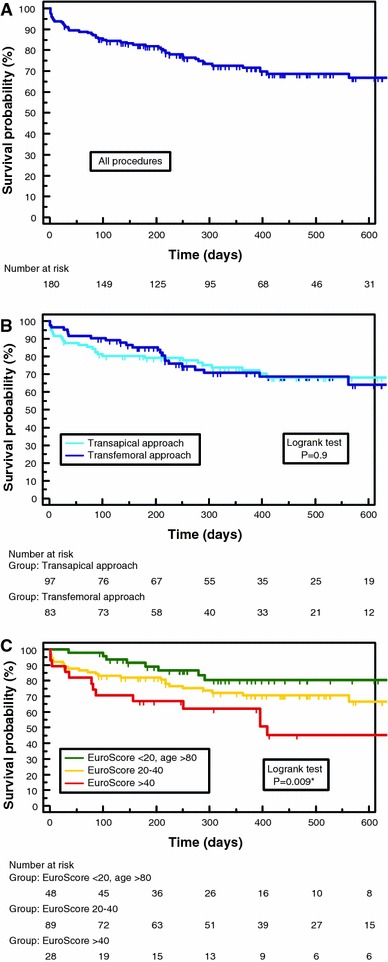
All-cause mortality Kaplan–Meier survival during follow-up in **a** all 180 patients treated with TAVI, **b** patients after transapical or transfemoral procedures separated, and **c** three patient cohorts stratified according to preoperative logistic EuroScore mortality estimates

The telephone follow-up was 99.4% complete. Survival proportions at 6 and 12 months were 82 and 72%, respectively. Between the two approaches, no significant differences could be observed (*P* = 0.9). Figure 2a illustrates the Kaplan–Meier survival after percutaneous valve implantation in the whole cohort and 2b for both approaches separated.

At a median follow-up of 319 days, 51 of all 180 patients (28.3%) had died. The causes of death (*n* = 33) occurring after discharge from the index hospitalization were the following: unexplained sudden deaths (*n* = 8); congestive heart failure (*n* = 5); device endocarditis (*n* = 2); stroke (*n* = 1); pneumonia or other septicaemia (*n* = 4); cancer (*n* = 1); surgery after hip fracture (*n* = 1); ileus of small intestine (*n* = 1); old age and bad clinical condition (*n* = 10). Altogether, 16 deaths (48%) had to be counted as cardiovascular.

### Mid-term outcome—event-free survival

**Fig. 3:**
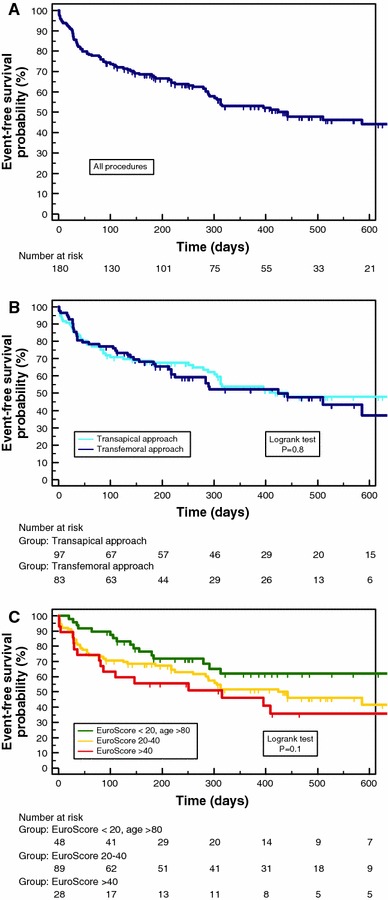
Event-free survival Kaplan–Meier curves of event-free survival during follow-up in **a** all 180 patients treated with TAVI, **b** patients after transapical or transfemoral procedures separated, and **c** three patient cohorts stratified according to preoperative logistic EuroScore mortality estimates

Furthermore, the occurrence of major adverse cardiovascular events during follow-up was explored (see Fig. 3a). Reasons for further hospitalization were the following (one patient could have more than one event): congestive heart failure (*n* = 26), stroke (*n* = 9), myocardial infarction (*n* = 4), access complications of TAVI procedure occurring after first discharge (*n* = 4), syncope requiring pacemaker implantation (*n* = 3), and aortic valve-related events (2 reinterventions, 5 cases of suspected device endocarditis). Survival curves (*P* = 0.8) as well as observed median event-free survival (424 days in the transfemoral and 442 days in the transapical group) did not differ significantly between both procedures (see Fig. 3b).

**Fig. 4:**
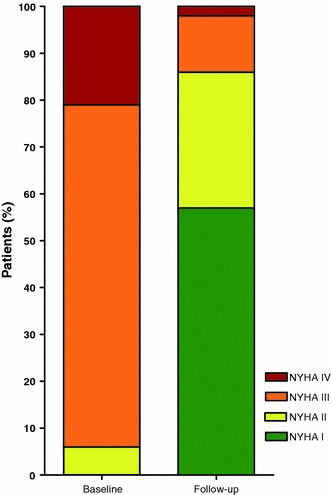
NYHA-status before TAVI and at time of telephone follow-up

Whereas 94% of patients had presented with dyspnoea of NYHA functional classes III and IV at baseline, 57% had no and 29% only mild dyspnoea (NYHA II) at follow-up. However, 12% still complained of dyspnoea NYHA class III and 2% of dyspnoea at rest after TAVI (see Fig. 4).

## Discussion

### Patient characteristics

Regarding the total cohort, our patient population is comparable to that of many other publications concerning age, comorbidities and logistic EuroScore [3, 11–18]. However, in contrast to other studies, logistic EuroScore and most other baseline parameters did not differ significantly between transapical and transfemoral patients. We attribute this fact to our previously described treatment strategy allocating a significant proportion of higher risk patients to the transfemoral approach to avoid general anaesthesia.

### Early outcome and periprocedural complications

Procedural safety and outcome of TAVI at our institution are not inferior to that of other documented series [3, 11–19]. Procedural success was very high (96.7%). The 30-day mortality (as-treated analyses) of 8.9% was well in line with the one observed in other series ranging from 5.2 [3] to 12.4% [18]. However, we could not demonstrate a significant difference in 30-day mortality between transapical and transfemoral procedures (12.4 vs. 4.8%, *P* = 0.08). Comparing exclusively Edwards implantations in TA and TF approaches, no statistical significant difference could be observed either (12.4 vs. 5.1%, *P* = 0.13). In addition, we saw no significant effect of the learning curve: 30-day mortality did not differ between the first and the second half of implantations in all procedures combined (*P* = 0.6), in transfemoral approaches (*P* = 1.0) or in transapical approaches (*P* = 0.6).

The correlation between postprocedural worsening of renal function and morbidity as well as mortality is well documented. We observed a high incidence of stage 3 AKI (16%), and in 15% renal replacement therapy was required. Of note, the incidence of both events was significantly higher after transapical procedures (25 vs. 6% and 22 vs. 5%, *P* = 0.001) in spite of comparable baseline serum creatinine levels. Regarding exclusively TA patients, persons with AKI stage III had significantly lower minimum haemoglobin values after TAVI and received significantly more red blood cell transfusions than individuals with lower AKI stages. For example, in patients without AKI (*n* = 22) haemoglobin fell to a minimum of 9.3 ± 1.1 g/dl and 0.3 ± 0.7 RBC units were administered, whereas in stage-III AKI patients (*n* = 24) minimum haemoglobin was 8.2 ± 1.0 g/dl (*P* = 0.0004) and 3.0 ± 2.9 RBC units were given (*P* < 0.0001). These findings suggest that severity of AKI in transapical procedures depends on the extent of post-operative anaemia. A similar significant correlation was not present in TF patients. It can be hypothesized that the combination of peripheral vascular disease (which was significantly more frequent in TA patients) and severe anaemia might be responsible for the higher extent of renal damage after TA TAVI.

Access complications are a major drawback of TAVI procedures, with consequent cases of death in several studies. In our cohort, we experienced major vascular complications in 24% of transfemoral procedures. However, only one death occurred due to vascular damage (thoracic aortic dissection), indicating that the team was well prepared to manage these potentially life-threatening events. Other publications using VARC or comparable definitions reported major vascular complications in 16.4 [14] and 16.2% [15] of TF patients, respectively. After the performance of the first 30 cases of overall 59 transfemoral implantations of Edwards devices in our department, the introduction of the smaller Novaflex catheter as well as growing experience led to a significant decrease of major vascular complications following these procedures (from 37 to 14%, *P* = 0.04).

The significantly higher rate of new-onset left bundle branch block and pacemaker implantations after CoreValve procedures has been consistently documented in many previous studies and is attributed to the deeper intraventricular insertion of this device. However, the indication for postprocedural pacemaker implantations was overall relatively low in our patients (5% in the whole cohort, 17% for CoreValve) compared to other publications reporting rates between 3.8% (exclusively Edwards prosthesis implanted) [3] and 39.3% (84% CoreValve devices implanted) [18].

### Mid-term survival and morbidity

We reported a 12-month survival of 72% in our cohort, which compares favourably to data from other series [3, 11, 13, 15, 20] ranging between 69 [15] and 78% [13]. Recently, a 1-year survival of 76.5% was reported for a great patient cohort (*n* = 1506) in the SOURCE Registry [17]. Of note, we observed no difference in 1-year survival proportions between TA and TF patients (72 vs. 71%, *P* = 0.9).

To date, information on further hospitalizations and incidence of MACCE during follow-up in patients after TAVI is still limited. In our study, we reported a 1-year event-free survival of 53% in the whole group which compares favourably to the findings of Leon et al. (42.5% of their patients had reached the composite end point of death from any cause or repeat hospitalization at 1 year) [15]. However, event-free-survival in these elderly patients with significant comorbidities is relatively poor. In our patients, persisting congestive heart failure was the leading reason for further hospitalization (16%), and a frequent cause of death during follow-up (3%). Dysfunction of the prosthetic valve (major paravalvular leak) was present in two TF patients necessitating reinterventions, but could be excluded in the majority of cases. In these patients, cardiac decompensation was attributed either to ischaemic heart disease, severe mitral regurgitation or severe diastolic dysfunction due to persisting left ventricular hypertrophy. In summary, follow-up morbidity seems to be substantially determined by *cardiac* comorbidities.

### Subgroup analysis of mid-term outcome

We further stratified all-cause mortality as well as event-free survival by preoperative logistic EuroScore (with cut-offs basing on recommended indications for TAVI [4, 5]) creating three subgroups of patients: EuroScore >40% (*n* = 28), 20–40% (*n* = 89), and <20%. In the latter group, very advanced age (>80 years) represented the principal reason for the heart team’s preference of TAVI over conventional surgery (*n* = 48). To define a consistent subgroup, the remaining 15 patients with EuroScore <20% (including 4 deaths) were not included in the following analysis because of very heterogeneous comorbidities justifying their treatment with TAVI. However, their inclusion would not have changed the statistical result. Figure 2c demonstrates the survival curves of the three defined subgroups which differed significantly (*P* = 0.009). One-year survival proportions were 62% in patients with a preoperative logistic EuroScore of >40% (observed median survival of 409 days), 71% in patients with EuroScore 20–40%, and 80% in octogenarians with EuroScore <20%. Regarding event-free survival as an indicator for morbidity, we obtained similar even though not statistically significant results (*P* = 0.1). Patients with a logistic EuroScore >40% had a 1-year event-free survival of 46%, in contrast to 52% in the second and 62% in the third group (Fig. 3c). Observed median event-free survival was 315, 442 and 710 days in the previously defined subgroups.

In conclusion, the stratification by preoperative logistic EuroScore (which is commonly used as a tool to predict 30-day mortality) allowed a convincing 1-year survival prognosis in our cohort, whereas the stratification by approach did not. Our results are suggesting that mortality and event-free survival do not primarily depend on the type of approach, but rather on the incidence of comorbidities.

In Europe, the logistic EuroScore has been widely accepted by cardiologists and cardiac surgeons as a “gold standard” [21] to aquire a general idea of peri-operative risk. In recent years it has become evident that it overestimates in-hospital mortality and that its discriminatory ability in patients undergoing aortic valve surgery is worse than in patients with isolated coronary surgery [22, 23]. Therefore, Dewey et al. [24] suggested the Society of Thoracic Surgeons (STS) Score as the most reliable single risk scoring model for both peri-operative mortality and long-term survival after isolated AVR in extremely high-risk patients. However, the STS score is much more sophisticated than the logistic EuroScore complicating its routine use in daily clinical practice. Furthermore, Leontyev et al. [25] found that stratification of octogenarians undergoing surgical AVR by logistic EuroScore revealed no significant differences in peri-operative outcomes, but proved good at differentiating survival during medium-term follow-up. The utility of both surgical prediction scores in the context of TAVI is frequently questioned. However, in our opinion the strength of scoring systems is to provide some kind of risk stratification for different patient cohorts (regardless of the calculated absolute value), whereas they are unsuitable for the prediction of an individual patient’s risk and cannot replace clinical judgement as the crucial factor of medical decision-making.

But does the performance of TAVI procedures in otherwise relatively healthy octogenarians fulfil the condition of “off-label-use”? Other TAVI series included up to 60% of patients with a logistic EuroScore <20% [18], and our own cohort compares favourably to the Source Registry [16] which consists to one-third of such patients. According to current apprehensions, the rapid spread of the TAVI technique carries the danger of withdrawing good surgical candidates from conventional aortic valve replacement. However, in our opinion this reproach would not be appropriate concerning our cohort of octogenarians characterized by a mean age of 84 ± 3 years and a mean logistic EuroScore of 14 ± 3%. As we learned from the study of Iung et al. [2], surgery is denied in at least one-third of elderly patients with symptomatic AS, and “advanced age” represents the main reason for denial of surgery. Opponents of TAVI could argue that conventional AVR is feasible in octogenarians. Previously reported in-hospital mortality rates [26–28] ranged between 4.5 [28] and 9% [27] in selected octogenarians (logistic EuroScore not reported). Lately, the results of the PARTNER trial [3] demonstrated a mortality rate of 6.5% after surgical AVR in patients randomized for either TAVI or surgery. However, in our own specific patient cohort, in-hospital mortality was 2% with just one case of death. In addition, in this elderly population not only mortality but also morbidity after surgery is an important consideration. Ben-Dor et al. [29] reported a significant reduction of quality of life in one-fifth of elderly patients after surgical AVR, and some even lost their independence. Furthermore, prolonged hospital stays (>14 days) following this intervention were reported in up to 50% of elderly patients [26]. Finally, concerns of device durability do not have a high priority in octogenarians, and Gurvitch et al. [11] already demonstrated the absence of relevant device deterioration in a period up to 3 years.

According to our data, octogenarians with logistic EuroScore <20% seem to be acceptable candidates for TAVI procedures with good mid-term survival and reasonably low morbidity. In contrast, patients with logistic EuroScore >40% do poorly and survive on average little more than 1 year, suggesting that indication for TAVI and risk-to-benefit ratio should be validated carefully in each single case.

## Conclusions

Procedural safety and outcome data of TAVI in high-risk patients are convincing, although TAVI unquestionably remains a highly invasive procedure. The widespread used logistic EuroScore for the prediction of peri-interventional mortality rather reflects the 1-year mortality in our patients. Concerning the type of approach, bleeding complications occurred more frequently in the TF and worsening of renal function more often in the TA group, but survival and rehospitalization rates did not differ significantly. In our opinion, neither procedure is per se superior to the other, but the type of approach should be carefully allocated according to the patients’ individual comorbidities.

However, mid-term mortality and rate of rehospitalization after TAVI are considerably high in specific patient cohorts, suggesting that selection criteria for the identification of patients who would benefit most from TAVI procedures need to be refined. In the present study, octogenarians with logistic EuroScore <20% could be identified as candidates with good mid-term survival and relatively low morbidity.

## References

[1] Vahanian A, Baumgartner H, Bax J, Butchart E, Dion R, Filippatos G, Flachskampf F, Hall R, Iung B, Kasprzak J, Nataf P, Tornos P, Torracca L, Wenink A (2007). Guidelines on the management of valvular heart disease: the Task Force on the management of valvular heart disease of the European Society of Cardiology. Eur Heart J.

[2] Iung B, Cachier A, Baron G, Messika-Zeitoun D, Delahaye F, Tornos P, Gohlke-Barwolf C, Boersma E, Ravaud P, Vahanian A (2005). Decision-making in elderly patients with severe aortic stenosis: why are so many denied surgery?. Eur Heart J.

[3] Smith CR, Leon MB, Mack MJ, Miller DC, Moses JW, Svensson LG, Tuzcu EM, Webb JG, Fontana GP, Makkar RR, Williams M, Dewey T, Kapadia S, Babaliaros V, Thourani VH, Corso P, Pichard AD, Bavaria JE, Herrmann HC, Akin JJ, Anderson WN, Wang D, Pocock SJ (2011). Transcatheter versus surgical aortic-valve replacement in high-risk patients. N Engl J Med.

[4] Vahanian A, Alfieri O, Al-Attar N, Antunes M, Bax J, Cormier B, Cribier A, De JP, Fournial G, Kappetein AP, Kovac J, Ludgate S, Maisano F, Moat N, Mohr F, Nataf P, Pierard L, Pomar JL, Schofer J, Tornos P, Tuzcu M, van Hout B, Von Segesser LK, Walther T (2008). Transcatheter valve implantation for patients with aortic stenosis: a position statement from the European Association of Cardio-Thoracic Surgery (EACTS) and the European Society of Cardiology (ESC), in collaboration with the European Association of Percutaneous Cardiovascular Interventions (EAPCI). Eur Heart J.

[5] Figulla HR, Cremer J, Walther T, Gerckens U, Erbel R, Osterspey A, Zahn R (2009). Positionspapier zur kathetergeführten Aortenklappenintervention. Kardiologe.

[6] Grube E, Laborde JC, Gerckens U, Felderhoff T, Sauren B, Buellesfeld L, Mueller R, Menichelli M, Schmidt T, Zickmann B, Iversen S, Stone GW (2006). Percutaneous implantation of the CoreValve self-expanding valve prosthesis in high-risk patients with aortic valve disease: the Siegburg first-in-man study. Circulation.

[7] Lichtenstein SV, Cheung A, Ye J, Thompson CR, Carere RG, Pasupati S, Webb JG (2006). Transapical transcatheter aortic valve implantation in humans: initial clinical experience. Circulation.

[8] Webb JG, Chandavimol M, Thompson CR, Ricci DR, Carere RG, Munt BI, Buller CE, Pasupati S, Lichtenstein S (2006). Percutaneous aortic valve implantation retrograde from the femoral artery. Circulation.

[9] Leon MB, Piazza N, Nikolsky E, Blackstone EH, Cutlip DE, Kappetein AP, Krucoff MW, Mack M, Mehran R, Miller C, Morel MA, Petersen J, Popma JJ, Takkenberg JJ, Vahanian A, van Es GA, Vranckx P, Webb JG, Windecker S, Serruys PW (2011). Standardized endpoint definitions for transcatheter aortic valve implantation clinical trials: a consensus report from the Valve Academic Research Consortium. Eur Heart J.

[10] Seipelt RG, Hanekop G, Schillinger W (2010). Migration of a transcatheter aortic valve in the left ventricular outflow tract. Heart.

[11] Gurvitch R, Wood DA, Tay EL, Leipsic J, Ye J, Lichtenstein SV, Thompson CR, Carere RG, Wijesinghe N, Nietlispach F, Boone RH, Lauck S, Cheung A, Webb JG (2010). Transcatheter aortic valve implantation: durability of clinical and hemodynamic outcomes beyond 3 years in a large patient cohort. Circulation.

[12] Eltchaninoff H, Prat A, Gilard M, Leguerrier A, Blanchard D, Fournial G, Iung B, Donzeau-Gouge P, Tribouilloy C, Debrux JL, Pavie A, Gueret P (2011). Transcatheter aortic valve implantation: early results of the FRANCE (FRench Aortic National CoreValve and Edwards) registry. Eur Heart J.

[13] Himbert D, Descoutures F, Al-Attar N, Iung B, Ducrocq G, Detaint D, Brochet E, Messika-Zeitoun D, Francis F, Ibrahim H, Nataf P, Vahanian A (2009). Results of transfemoral or transapical aortic valve implantation following a uniform assessment in high-risk patients with aortic stenosis. J Am Coll Cardiol.

[14] Lefevre T, Kappetein AP, Wolner E, Nataf P, Thomas M, Schachinger V, De BB, Eltchaninoff H, Thielmann M, Himbert D, Romano M, Serruys P, Wimmer-Greinecker G (2011). One year follow-up of the multi-centre European PARTNER transcatheter heart valve study. Eur Heart J.

[15] Leon MB, Smith CR, Mack M, Miller DC, Moses JW, Svensson LG, Tuzcu EM, Webb JG, Fontana GP, Makkar RR, Brown DL, Block PC, Guyton RA, Pichard AD, Bavaria JE, Herrmann HC, Douglas PS, Petersen JL, Akin JJ, Anderson WN, Wang D, Pocock S (2010). Transcatheter aortic-valve implantation for aortic stenosis in patients who cannot undergo surgery. N Engl J Med.

[16] Thomas M, Schymik G, Walther T, Himbert D, Lefevre T, Treede H, Eggebrecht H, Rubino P, Michev I, Lange R, Anderson WN, Wendler O (2010). Thirty-day results of the SAPIEN aortic Bioprosthesis European Outcome (SOURCE) Registry: a European registry of transcatheter aortic valve implantation using the Edwards SAPIEN valve. Circulation.

[17] Thomas M, Schymik G, Walther T, Himbert D, Lefevre T, Treede H, et al. (2011) 1-year results from combined cohort I and cohort II of the SOURCE registry

[18] Zahn R, Gerckens U, Grube E, Linke A, Sievert H, Eggebrecht H, Hambrecht R, Sack S, Hauptmann KE, Richardt G, Figulla HR, Senges J (2011). Transcatheter aortic valve implantation: first results from a multi-centre real-world registry. Eur Heart J.

[19] Figulla L, Neumann A, Figulla HR, Kahlert P, Erbel R, Neumann T (2010). Transcatheter aortic valve implantation: evidence on safety and efficacy compared with medical therapy. A systematic review of current literature. Clin Res Cardiol.

[20] Webb JG, Altwegg L, Boone RH, Cheung A, Ye J, Lichtenstein S, Lee M, Masson JB, Thompson C, Moss R, Carere R, Munt B, Nietlispach F, Humphries K (2009). Transcatheter aortic valve implantation: impact on clinical and valve-related outcomes. Circulation.

[21] Bode C, Kelm M (2009). EUROSCORE: still gold standard or less?. Clin Res Cardiol.

[22] Grossi EA, Schwartz CF, Yu PJ, Jorde UP, Crooke GA, Grau JB, Ribakove GH, Baumann FG, Ursumanno P, Culliford AT, Colvin SB, Galloway AC (2008). High-risk aortic valve replacement: are the outcomes as bad as predicted?. Ann Thorac Surg.

[23] Gummert JF, Funkat A, Osswald B, Beckmann A, Schiller W, Krian A, Beyersdorf F, Haverich A, Cremer J (2009). EuroSCORE overestimates the risk of cardiac surgery: results from the national registry of the German Society of Thoracic and Cardiovascular Surgery. Clin Res Cardiol.

[24] Dewey TM, Brown D, Ryan WH, Herbert MA, Prince SL, Mack MJ (2008). Reliability of risk algorithms in predicting early and late operative outcomes in high-risk patients undergoing aortic valve replacement. J Thorac Cardiovasc Surg.

[25] Leontyev S, Walther T, Borger MA, Lehmann S, Funkat AK, Rastan A, Kempfert J, Falk V, Mohr FW (2009). Aortic valve replacement in octogenarians: utility of risk stratification with EuroSCORE. Ann Thorac Surg.

[26] Kolh P, Kerzmann A, Lahaye L, Gerard P, Limet R (2001). Cardiac surgery in octogenarians; peri-operative outcome and long-term results. Eur Heart J.

[27] Kolh P, Kerzmann A, Honore C, Comte L, Limet R (2007). Aortic valve surgery in octogenarians: predictive factors for operative and long-term results. Eur J Cardiothorac Surg.

[28] Ngaage DL, Cowen ME, Griffin S, Guvendik L, Cale AR (2008). Are initial valve operations in octogenarians still high-risk in the current era?. J Heart Valve Dis.

[29] Ben-Dor I, Pichard AD, Gonzalez MA, Weissman G, Li Y, Goldstein SA, Okubagzi P, Syed AI, Maluenda G, Collins SD, Delhaye C, Wakabayashi K, Gaglia MA, Torguson R, Xue Z, Satler LF, Suddath WO, Kent KM, Lindsay J, Waksman R (2010). Correlates and causes of death in patients with severe symptomatic aortic stenosis who are not eligible to participate in a clinical trial of transcatheter aortic valve implantation. Circulation.

